# Epididymal Adenomatoid Tumour: A Case Report

**DOI:** 10.7759/cureus.47505

**Published:** 2023-10-23

**Authors:** Mohamed Farah, Mosea Song, Wasim Mahmalji

**Affiliations:** 1 Urology, Wye Valley NHS Trust, Hereford, GBR

**Keywords:** immunohistochemical features, para-testicular tumours, testicular neoplasm, epididymis, adenomatoid tumour

## Abstract

Adenomatoid tumours are rare benign neoplasm involving the para testicular region, mostly the tail of the epididymis. They are typically small, firm and asymptomatic masses in the scrotal region and often discovered incidentally during physical examination or imaging studies. It is very challenging to differentiate them clinically and radiologically from malignant intratesticular solid tumours, which may lead to unnecessary orchidectomies. This case report presents the clinical management of a 57-year-old male patient with adenomatoid tumour of the epididymis, highlighting the diagnostic workup, surgical approach and postoperative outcomes. In addition, a comprehensive literature review was conducted to discuss the morphological and immunohistochemical features to improve understanding of these rare lesions and assist in accurate diagnosis and appropriate management.

## Introduction

Paratesticular tumours are rare and accounts for less than 5% of all intrascrotal solid tumours [1]. Epididymal tumours are a rare subtype of paratesticular neoplasms with the vast majority of these tumours exhibiting benign behaviour [2]. Adenomatoid neoplasms represent the most common histological variant followed by leiomyoma and then papillary cystadenoma [3]. Diagnosing epididymal adenomatoid tumours can be challenging, as clinical and sonographic features are not conclusive. Histological confirmation after surgical excision is required for an accurate diagnosis. Herein, we describe a case of an epididymal adenomatoid tumour in a 57-year-old patient that presented with a scrotal mass, which was managed with limited surgical excision, and diagnosis was confirmed by histopathological analysis of the resected lesion.

## Case presentation

A 57-year-old man presented to the urology department with a three-month history of progressive painless right-sided testicular nodule. Medical and surgical examinations were unremarkable with no history of epididymits or trauma.

Physical examination showed a round, firm, painless and hard intrascrotal mass inferior to the right testicule, which was distinct from the testicle and arising from the surface of the epididymis. The overlying skin, epididymis, spermatic cord and left testicule were normal, and the inguinal lymph nodes were not palpable.

**Figure 1:**
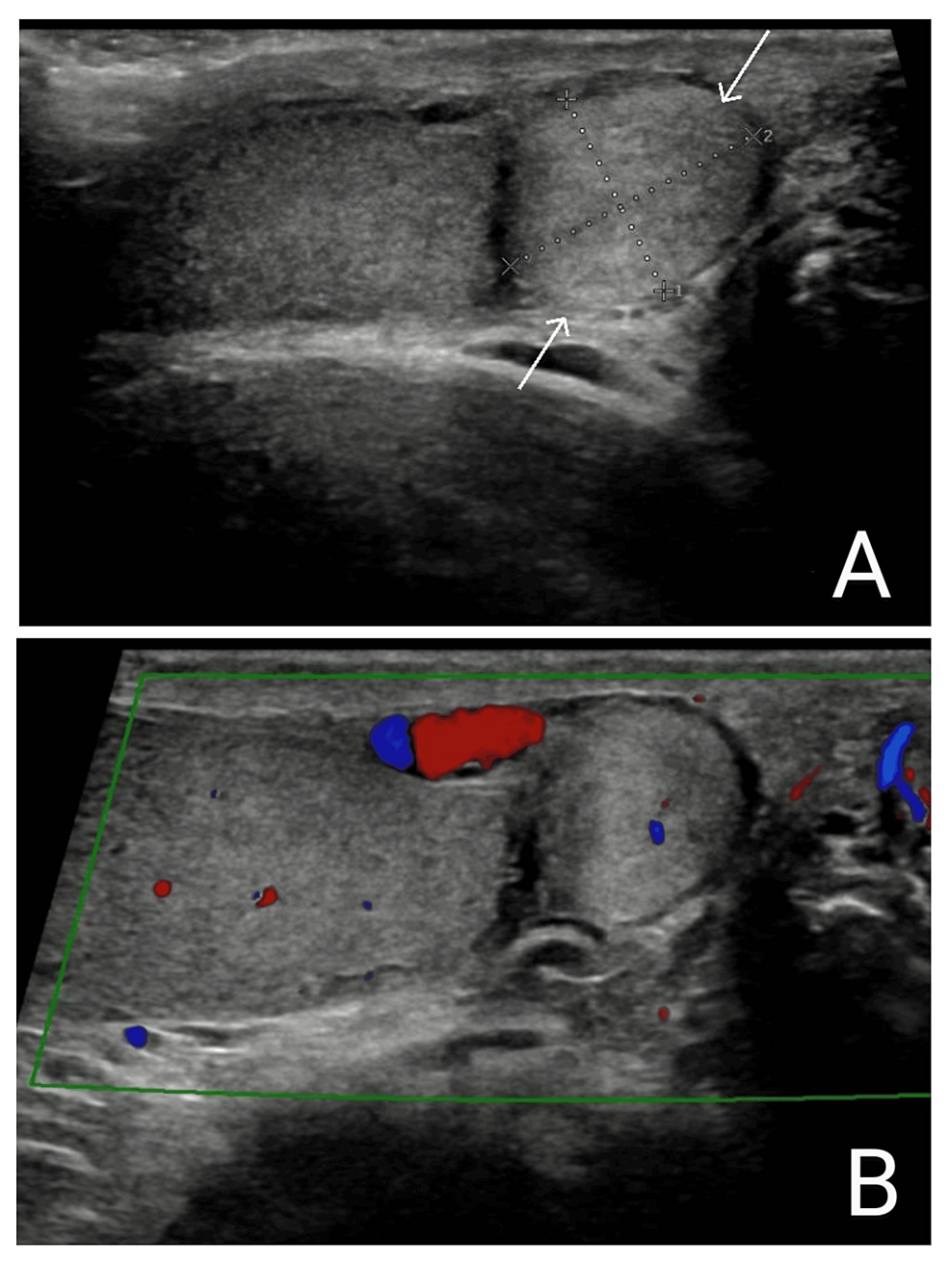
Epididymal adenomatoid tumour. Longitudinal grayscale (A) and color flow Doppler (B) ultrasounds of the scrotum reveals a well-defined solid mass (within arrows), measuring 12 x 10 mm in size and arising from the right epididymal tail without any disruption of the architecture of the testical parenchyma. The lesion showed minimal internal vascularity and slightly increased echogenicity compared to the testicular parenchyma.

Scrotal ultrasonography revealed a well-defined solid rounded mass measuring about 12 x 10 mm in size and arising from the right epididymal tail without any disruption of the architecture of the testical parenchyma. The lesion showed minimal internal vascularity and slightly increased echogenicity compared to the testicular parenchyma immediately adjacent to the mass (Figure 1). Laboratory investigations, including alpha-fetoprotein (AFP), beta-human chorionic gonadotropin (β-hCG) and lactate dehydrogenase (LDH), were all within normal ranges.

The case was discussed in a multidisciplinary team meeting (MDT), leading to the decision to carry out an epididemectomy due to the strong suspicion of a benign tumour based on the presentation and clinical and radiological findings. Subsequently, the patient underwent a right-sided scrotal exploration, which revealed a greyish-white, hard, well-circumscribed nodule, located on the tail of the right epididymis without involvement of the testicular parenchyma. A testicular-preserving surgery was performed in the form of an epididymectomy, and the mass was excised without any damage to adjacent structures.

**Figure 2:**
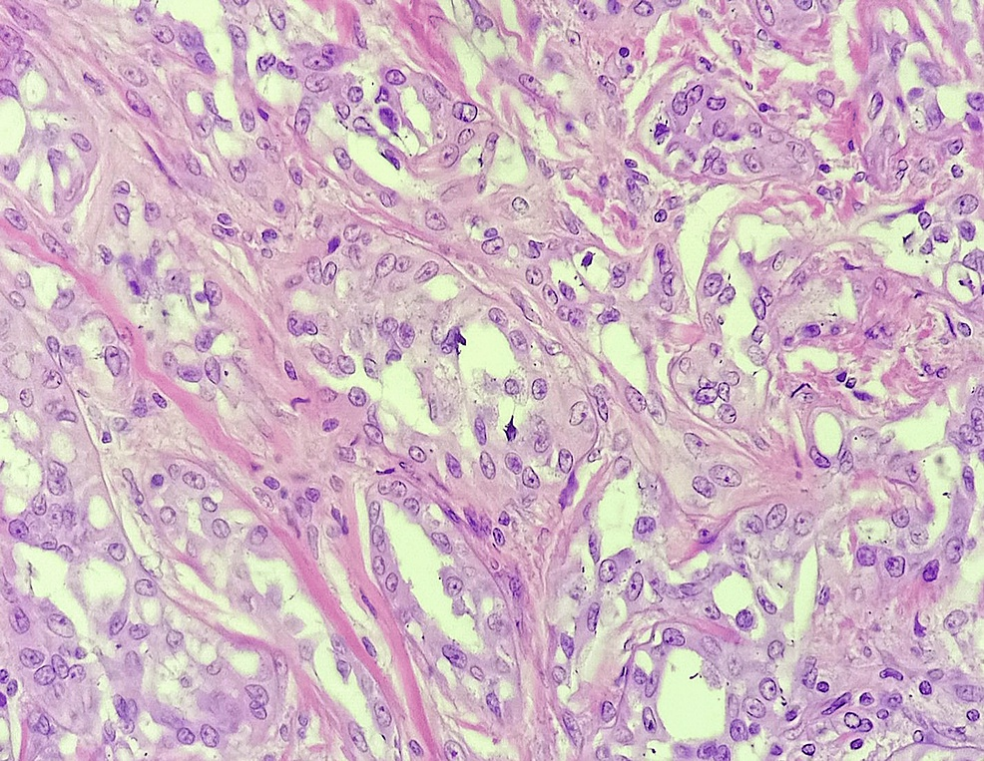
Histopathology of the epididymal mass. Histopathological image of the excised mass demonstrating tubules and cord of tumour cells along with a large vaculated cytoplasm, which are characteristic for an adenomatoid tumour.

Histopathological examination of the excised nodule revealed cells arranged in compressed interconnected cords and tubules, surrounded by fibrous stroma and lined with cuboidal cells, with vacuolated cytoplasm (Figure 2). Immunohistochemical examination was positive for tumour markers calretinin, CK5/6 and WT-1, which confirm the diagnosis of adenomatoid tumour of the epididymis and its mesothelial origin (Table 1). One-year post-operative follow-up revealed no recurrence.

**Table 1:** Immunohistochemical staining results This staining profile, in conjunction with the histological features, established the diagnosis of an adenomatoid tumour.

Immunohistochemistry findings	
Stain	Results
Calretinin	+++
CK5/6	+++
WT-1	++

## Discussion

Para-testicular tumours are rare and comprise less than 5% of all intrascrotal solid tumours. An adenomatoid tumour is a distinctively rare benign neoplasm thought to be of mesothelial origin. Adenomatoid tumours are responsible for approximately 30% of all paratesticular masses [1,3]. Majority of adenomatoid tumours in males arise from the epididymis [4]. They occur more often in the tail than in the head of the epididymis and rarely involve other areas of the male genitalia, such as the testis, tunica albuginea, spermatic cord, ejaculatory ducts, prostate and suprarenal recess. These tumours can also occur in other areas of the male genitalia, such as the spermatic cord, prostate and ejaculatory ducts, although they are less common in these locations [4,5].

Epididymal adenomatoid tumours are most commonly found in middle-aged patients, with an average age of clinical presentation ranging from 32 to 66 years [2]. Most adenomatoid tumours of the epididymis are asymptomatic and are discovered incidentally during routine physical examination, as a non-painful scrotal mass more commonly located at the tail of the epididymis [6]. In some cases, patients may present with symptoms, such as chronic scrotal pain or swelling [7]. However, these symptoms are not specific to epididymal adenomatoid tumours and can be seen in other conditions as well. On physical examination, a well-defined, rounded lesion can be found in the tail of the epididymis.

The histological origin of epididymal adenomatoid tumours is believed to be of mesothelial origin. Sangoi et al. conducted a clinicopathological and immunohistochemical study of 44 cases of adenomatoid tumours of the female and male genital tracts [2]. They found that adenomatoid tumours exhibited a mesothelial phenotype based on immunohistochemical staining patterns [2]. These findings strongly support a mesothelial origin for adenomatoid tumours.

It is important to consider the differential diagnosis when evaluating epididymal lesions. Conditions that can mimic epididymal adenomatoid tumours include spermatocele, testicular germ cell tumours, mesothelioma and other benign or malignant neoplasms [2]. A comprehensive analysis of clinical, histopathological and immunohistochemical features can help differentiate epididymal adenomatoid tumours from these conditions.

The diagnosis of epididymal adenomatoid tumours can be challenging due to their rarity and similarity to other benign and malignant tumours. Ultrasonography (US) of the testes is the method of choice for diagnosing scrotal pathology and is often performed as a part of preoperative investigation given its accessibility and cost-effectiveness. The sonographic findings of adenomatoid tumours of the epididymis typically include a solid well-defined extratesticular mass and varies from hypoechoic to isoechoic to hyperechoic compared with adjacent tissues [8]. These tumours often have a homogeneous echotexture and may show increased vascularity on Doppler imaging. However, it is important to note that these ultrasound findings are not specific to epididymal adenomatoid tumours and can also be observed in other epididymal lesions [8]. Although not always necessary, magnetic resonance imaging (MRI) should be performed when the US findings are unclear regarding the tumour boundaries and its local invasiveness or when the mass originates from the tunica albuginea. In those cases, MRI can identify the margins separating the tumour from the testicular parenchyma [9].

If there is a high suspicion of malignancy based on pre-operative findings, excision of the epididymal tumour is performed after the inguinal approach [10]. Otherwise, the approach can be scrotal [10]. Surgical excision with testis-sparing surgery, in cases of low suspicion of malignancy, is the recommended management approach for epididymal adenomatoid tumours [11]. As the most common paratesticular tumour is benign in nature, this approach allows for the removal of the tumour while preserving the testis and avoiding unnecessary orchidectomy [11]. If separating the mass from the adjacent structures proves challenging, an intraoperative biopsy should be performed. Since there have been no reported cases of post-operative recurrence, total tumour excision is regarded as curative [6,7,12]. In our case, a benign tumour was strongly suspected based on the presentation and clinical and radiological findings, so a trans-scrotal approach with mass removal was performed.

Microscopic features of adenomatoid tumour include epithelial-like cells forming solid cords, tubules and cyst coated by a layer of flat or cuboidal epithelial cells. The main feature is the presence of vacuolated cytoplasm inside the cell. The stroma usually is fibrous and contain smooth muscle cells [13].

Immunohistochemically, an adenomatoid tumour is positive for markers, such as pancytokeratins, podoplanin, WT-1 and calretinin. Among these, calretinin is a mesothelial marker that shows strong positivity in adenomatoid tumours [14]. These markers are typically expressed in adenomatoid tumours and support their mesothelial origin [2,14]. Positive immunohistochemical stains for adenomatoid tumours can aid in distinguishing them from other neoplasms that may be mistaken for adenomatoid tumours, such as yolk sac tumour (which is negative for WT1 and calretinin), Leydig cell tumour (which is negative for WT1) and metastatic carcinoma. In addition, the exclusion of vascular origin tumours can be achieved by negative tests for epithelial markers, including factors VIII or CD34. Other tumour markers, such as alpha-fetoprotein (AFP), lactate dehydrogenase (LDH), carcinoembryonic antigen (CEA) and beta-human chorionic gonadotropin (b-HCG), when tested, are negative [15]. In our case, all the laboratory and histopathological markers confirmed the diagnosis of an adenomatoid tumour of the epididymis.

## Conclusions

Epididymal adenomatoid tumours are rare benign neoplasms that primarily affect the epididymis. Diagnosing these tumours can be challenging due to their rarity and the potential for mimicking other conditions. Testis-sparing surgery is the preferred treatment approach, and careful preoperative and intraoperative evaluation can help guide the surgical management of these tumours. Histological examination of excisional biopsy samples is required to confirm the diagnosis by identifying the characteristic histological features. Further research and case reports are needed to better understand the characteristics and optimal management of epididymal adenomatoid tumours.
